# Phytohormonal Regulation of Plant Responses to Major Abiotic Stresses: From Signaling Pathways to Hormonal Crosstalk

**DOI:** 10.3390/metabo16060401

**Published:** 2026-06-09

**Authors:** Shadi Sadat Mehrabi, Manijeh Sabokdast, Beata Dedicova

**Affiliations:** 1 Department of Agronomy and Plant Breeding, College of Agriculture and Natural Resources, University of Tehran, P.O. Box 4111, Karaj 31587-11167, Iran; shadi.mehrabi@ut.ac.ir; 2 Department of Plant Breeding, Swedish University of Agricultural Sciences (SLU), Alnarp, Sundsvägen 10, SE-234 22 Lomma, Sweden

**Keywords:** abiotic stress, phytohormones, hormonal crosstalk, extreme temperatures, strigolactones

## Abstract

Plants are constantly exposed to diverse abiotic stresses, including drought, salinity, and extreme temperatures, which severely limit growth, development, and crop productivity. These stresses disrupt physiological, biochemical, and molecular processes, leading to reduced photosynthesis, altered water and ion homeostasis, and accumulation of reactive oxygen species (ROS). Plants have evolved sophisticated sensing and signaling mechanisms to perceive these stresses, with phytohormones playing central roles in mediating adaptive responses. Key hormones, including abscisic acid (ABA), salicylic acid (SA), jasmonates (JAs), gibberellins (GAs), auxin (IAA), ethylene (ET), melatonin, and strigolactones (SLs), regulate stress tolerance by controlling stomatal behavior, root architecture, antioxidant systems, osmolyte accumulation, and stress-responsive gene expression. Importantly, these hormones operate within an intricate network of crosstalk, integrating multiple signaling pathways to balance growth and stress adaptation. Interactions among ABA, GA, JA, SA, auxin, ET, SLs, and melatonin enable plants to coordinate transcriptional regulation, protein phosphorylation, and ROS signaling, optimizing survival under fluctuating environmental conditions. Understanding the molecular mechanisms underlying hormonal crosstalk and their roles in abiotic stress tolerance provides valuable insights for developing resilient crops in the face of climate change.

## 1. Introduction

Plants are continuously exposed to a wide range of abiotic stresses, which are major constraints on crop growth and productivity worldwide [1]. Major abiotic stressors include drought, salinity, and extreme temperatures; collectively, they disrupt physiological and biochemical processes, leading to reduced biomass accumulation, impaired development, and lower yields [2,3]. Water deficit remains one of the most pervasive and damaging stresses affecting cultivated plants. Meta-analytical evidence indicates yield reductions of up to 50% under severe drought and water-limited conditions in major cereals [4]. Drought conditions, defined by sustained periods of below-normal soil moisture, limit plants’ access to water and constrain physiological processes such as photosynthesis and transpiration [5]. These limitations reduce leaf expansion, restrict cell division, and diminish overall plant vigor, especially during critical stages such as vegetative growth and reproductive development [6]. Moreover, drought often interacts with elevated temperatures to compound stress, further inhibiting water transport and metabolic balance [7]. Temperature extremes constitute another fundamental abiotic constraint. Heat stress occurs when ambient temperatures exceed levels conducive to normal biochemical and cellular activities, resulting in altered membrane fluidity, protein misfolding and denaturation, ROS accumulation, and induction of heat shock proteins (HSPs). These molecular and cellular disturbances impair enzymatic activities, destabilize cellular membranes, reduce photosynthetic efficiency, and ultimately shorten the photosynthetically effective period [8]. These effects are especially detrimental during the flowering and grain-filling stages, when reproductive success is highly temperature-sensitive [9]. In contrast, prolonged exposure to chilling or freezing conditions can cause extensive cellular damage and inhibit growth, ultimately reducing yield and product quality [10]. Salinity stress is increasingly problematic in many agricultural landscapes, especially in arid and semi-arid regions where irrigation practices exacerbate salt buildup [11]. High salt concentrations in the root zone increase soil osmotic potential, limiting water uptake and causing ionic imbalances within plant tissues. Excessive accumulation of sodium and chloride ions disrupts nutrient homeostasis and damages cellular structures, leading to reduced growth and productivity across a range of species [12]. Combined stresses, such as simultaneous drought and salinity, often cause more severe physiological disruptions than either stress alone.

Studies show that under combined stress, plants experience greater limitations in photosynthesis, ionic balance, and antioxidant capacity, underscoring the need for a deeper understanding of integrated stress responses in crop improvement programs [13]. Improving crop tolerance to abiotic stress remains a major challenge because many genes control stress tolerance [14]. Although conventional breeding has achieved significant progress in developing stress-tolerant cultivars in several crops, its efficiency is often constrained by the complex polygenic nature of stress responses and strong genotype–environment interactions [15,16,17]. As a result, there is still a need for complementary and sustainable strategies. Plants possess effective sensing and signaling systems that allow them to detect environmental stresses and adjust their growth and development accordingly. Among these systems, phytohormones play an important role as low-concentration signaling molecules that link environmental signals to internal developmental processes and help regulate plant growth, development, and stress responses [18].

Under abiotic stress, phytohormones regulate a wide range of plant responses at the physiological, biochemical, and molecular levels. These responses are mediated through complex intracellular signaling networks, including ROS, calcium (Ca^2+^) signaling, and protein kinase cascades such as mitogen-activated protein kinases (MAPKs) and SnRK2 kinases [19]. Phytohormones are also perceived through specific hormone receptor complexes, which initiate downstream signaling events that ultimately converge on transcription factors [20]. They are involved in processes such as stomatal control, changes in root growth and structure, activation of antioxidant systems, accumulation of osmolytes, and regulation of stress-related gene expression [21]. Through these interconnected signaling pathways, phytohormones help plants maintain cellular homeostasis and protect key physiological processes, such as photosynthesis, under stress conditions [22,23]. Importantly, phytohormone actions are not independent; instead, different hormones interact closely through signaling crosstalk. This interaction allows plants to fine-tune their stress responses [24].

This review comprehensively explores the roles of abscisic acid (ABA), salicylic acid (SA), jasmonates (JAs), gibberellins (GAs), auxin (IAA), ethylene (ET), melatonin, and strigolactones (SLs) in plant responses to abiotic stresses. In particular, it emphasizes how these phytohormones help maintain plant growth and productivity under unfavorable environmental conditions. By integrating current knowledge on hormonal crosstalk and signaling interactions, this work provides a more unified framework for understanding how multiple hormone pathways collectively regulate stress perception and adaptive responses.

## 2. Abiotic Stresses

### 2.1. Drought

**Figure 1:**
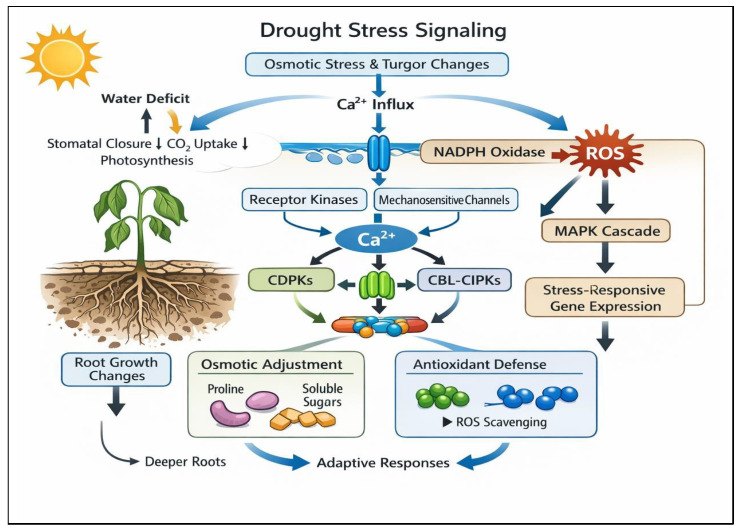
Molecular and physiological signaling networks underlying plant responses to drought stress. Water deficit induces osmotic stress and reduced cellular turgor, which are sensed by membrane-associated receptor-like kinases and mechanosensitive channels, triggering transient Ca^2+^ influx. Ca^2+^ signals are decoded by CDPKs and CBL–CIPKs, which regulate ion transport and stress-responsive gene expression [37]. Concurrently, activation of plasma membrane NADPH oxidases (RBOHs) increases ROS production, which serves as both a signaling molecule and a potential source of oxidative damage. ROS interacts with Ca^2+^ signaling and activates MAPK cascades, ultimately modulating transcriptional reprogramming [38,39,40]. Physiological adaptations include stomatal closure, reduced CO_2_ uptake and photosynthesis, osmotic adjustment through accumulation of compatible solutes such as proline and soluble sugars, activation of antioxidant defense systems, and modifications in root architecture to enhance water uptake [31].

Drought in plants includes a combination of interacting environmental and physiological factors such as prolonged soil water deficit that disturbs cellular water balance, high atmospheric evaporative demand (vapour pressure deficit), increased evapotranspiration, limitations in soil water-holding capacity, and the developmental stage of the plant, all of which collectively determine the severity of water stress and its impact on plant physiological processes including photosynthesis and transpiration [25,26]. Drought stress significantly reduces plant growth and crop productivity by creating a water deficit that disrupts cellular water balance [27]. Under drought, water loss triggers stomatal closure, which reduces transpiration but also restricts carbon dioxide (CO_2_) uptake, thereby lowering photosynthesis [28,29]. Water-deficit conditions lead to the accumulation of reactive oxygen species (ROS), which can damage cellular components, including membranes, proteins, and nucleic acids [30]. To adjust their internal water status, plants accumulate compatible solutes such as proline and soluble sugars, a process known as osmotic adjustment that helps maintain cell turgor under water-deficit conditions (Figure 1) [31].

Drought also induces changes in root architecture, including deeper or more extensive root growth, which improves water uptake from drier soil layers [32]. Drought stress rapidly activates signaling networks that control stress-responsive gene expression, with abscisic acid serving as a central regulator of this response [33]. At the early stages of drought perception, plants initiate rapid molecular signaling events that operate independently or partially independently of abscisic acid [34]. Changes in cellular turgor and osmotic pressure are sensed by membrane-associated receptor-like kinases and mechanosensitive channels, triggering transient increases in cytosolic Ca^2+^ [35,36]. These Ca^2+^ signatures are decoded by calcium-dependent protein kinases (CDPKs) and calcineurin B-like (CBL)-interacting protein kinases (CIPKs), which phosphorylate downstream targets to regulate ion transport and stress-responsive gene expression (Figure 1) [37]. Beyond potential cytotoxic effects, ROS act as second messengers that interact with Ca^2+^ signaling and activate mitogen-activated protein kinase (MAPK) cascades, thereby transmitting stress signals to the nucleus and modulating transcriptional reprogramming (Figure 1) [38,39,40].

### 2.2. Salinity

Salinity stress is one of the most pervasive abiotic stresses affecting agricultural productivity worldwide, arising primarily from the accumulation of soluble salts, especially sodium (Na^+^) and chloride (Cl^−^) ions, in the soil [41]. Excessive soil salinity significantly reduces plant growth and yield by imposing osmotic, ionic, and secondary oxidative stresses on cellular processes [42]. At the onset of salinity exposure, plants encounter osmotic stress, in which high external salt concentrations lower soil water potential, making water uptake more difficult and leading to reduced cell expansion and growth inhibition [43]. Over prolonged periods, elevated internal concentrations of Na^+^ and Cl^−^ cause ionic toxicity, disrupting nutrient balance and impairing essential physiological and cellular processes [44]. Salinity stress affects multiple key physiological and cellular processes. It reduces photosystem stability by impairing the efficiency of photosystems I and II, thereby decreasing photosynthetic performance. Stomatal conductance is often altered as plants regulate gas exchange to minimize water loss under osmotic stress [45]. Chloroplast ultrastructure is disrupted, including thylakoid membrane damage and pigment degradation, which further limits photosynthetic capacity [46]. Mitochondrial respiration is also affected, resulting in reduced ATP production and altered energy metabolism [47]. In addition, salinity stress disturbs Na^+^/K^+^ homeostasis by promoting excessive Na^+^ accumulation and inhibiting K^+^ uptake, which is critical for maintaining enzyme activity and cellular ionic balance [45].

**Figure 2:**
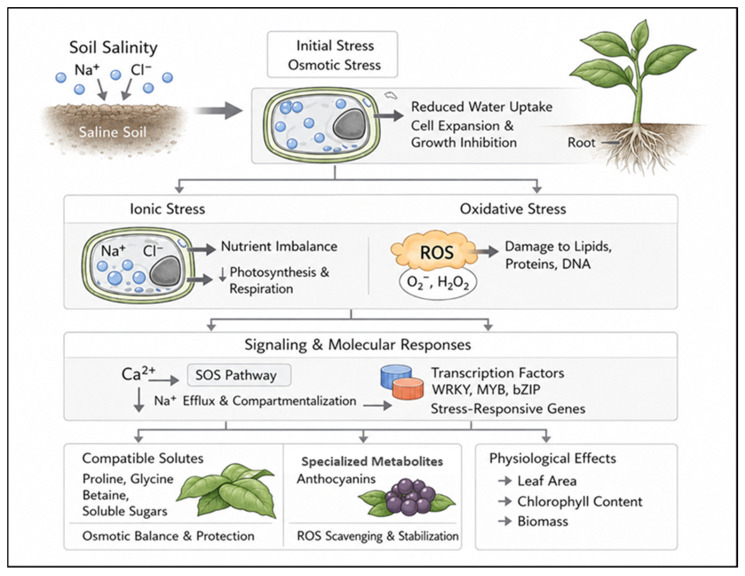
Plant responses to salinity stress. Soil salinity, primarily from Na^+^ and Cl^−^ accumulation, induces osmotic stress (reducing water uptake and cell expansion), ionic stress (nutrient imbalance and impaired photosynthesis and respiration), and oxidative stress (ROS accumulation) [48]. Stress perception triggers Ca^2+^-mediated SOS signaling and activates transcription factors (WRKY, MYB, bZIP) that regulate stress-responsive genes [51]. Adaptive responses include compatible solutes (proline, glycine betaine, sugars) and specialized metabolites (anthocyanins) that support osmotic balance and ROS scavenging, resulting in physiological effects such as reduced leaf area, chlorophyll content, and biomass [49,54].

Salinity also triggers the overproduction of reactive oxygen species (ROS), including superoxide radicals and hydrogen peroxide, which can damage lipids, proteins, and nucleic acids if not efficiently mitigated by antioxidant defense systems [48]. The combined effects of osmotic and ionic stresses often reduce leaf area, chlorophyll content, and overall biomass, serving as key physiological indicators of salt sensitivity in crop species [49]. Perception of salinity stress initiates complex signaling pathways that regulate stress-responsive gene expression. In plants, salt-induced increases in cytosolic Ca^2+^ act as a second messenger, activating protein kinase complexes, such as those in the salt overly sensitive (SOS) pathway, which maintain ion homeostasis by regulating Na^+^ efflux and vacuolar compartmentalization [50]. Transcription factors (TFs), including members of the WRKY, MYB, and basic leucine zipper (bZIP) families, are also activated under saline conditions and play crucial roles in orchestrating downstream gene expression involved in osmoprotection, antioxidant production, and stress adaptation (Figure 2) [51].

The synthesis of compatible solutes, such as proline, glycine betaine, and soluble sugars, is another important adaptive response observed in many species under salinity stress, helping maintain cellular osmotic balance and protect macromolecules [52,53]. Moreover, specialized metabolites such as anthocyanins accumulate in response to salinity, providing additional ROS-scavenging capacity and stabilizing cellular structures (Figure 2) [54].

### 2.3. Cold

**Figure 3:**
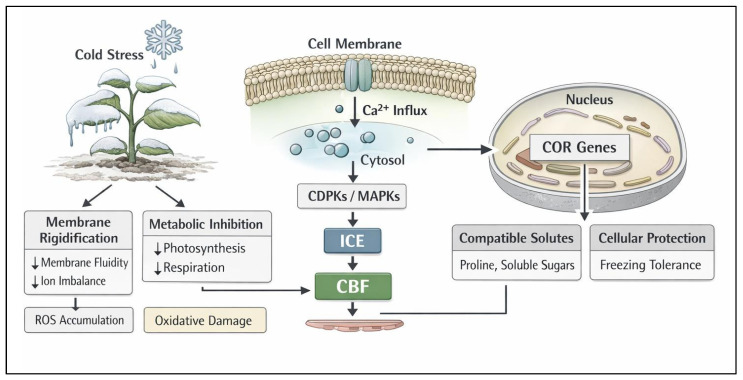
Mechanisms of the cold stress response in plants. Cold stress reduces membrane fluidity, disrupts ion homeostasis, inhibits metabolic enzymes, and induces ROS accumulation [58]. Membrane changes trigger Ca^2+^ influx, which activates CDPK and MAPK signaling pathways that regulate the ICE-CBF-COR transcriptional cascade. COR genes promote compatible solute accumulation and cellular protection, thereby enhancing freezing tolerance [61].

Cold stress is a major abiotic challenge for land plants because low temperatures disrupt normal cellular function and limit growth and productivity [55]. Under cold conditions, cellular membranes become less fluid. This change impairs membrane transport processes, including ion transport and aquaporin-mediated water movement, thereby disrupting nutrient distribution, water balance, and overall cellular stability [56]. Alongside membrane rigidification, key metabolic enzymes involved in photosynthesis and respiration show markedly reduced activity at low temperatures, thereby decreasing energy production and metabolic efficiency [57]. At the same time, cold stress increases the generation of reactive oxygen species. Although ROS are produced under normal conditions, their accumulation under stress can lead to oxidative damage to lipids, proteins, and nucleic acids if not adequately detoxified by antioxidant systems [58]. In response to these challenges, plants activate protective mechanisms that help mitigate damage and adapt to low-temperature conditions. One of the most consistent responses is the synthesis of compatible solutes such as proline and soluble sugars, which contribute to osmotic balance and protect cellular structures (Figure 3) [56].

Cold sensing in plant cells begins with changes in membrane physical properties that trigger an influx of Ca^2+^ into the cytosol. This rise in cytosolic Ca^2+^ acts as a second messenger, activating downstream signaling networks involving CDPKs and MAPKs that relay the stress signal toward the nucleus [59,60]. A central regulatory module in cold signaling is the Inducer of CBF Expression–C-repeat Binding Factor–Cold-Responsive (ICE–CBF–COR) cascade. ICE transcription factors initiate expression of CBF/dehydration-responsive element-binding (DREB) genes, which in turn activate cold-responsive (COR) genes. These COR genes enhance freezing tolerance by mediating the structural stabilization and metabolic reprogramming (Figure 3) [61].

### 2.4. Heat

**Figure 4:**
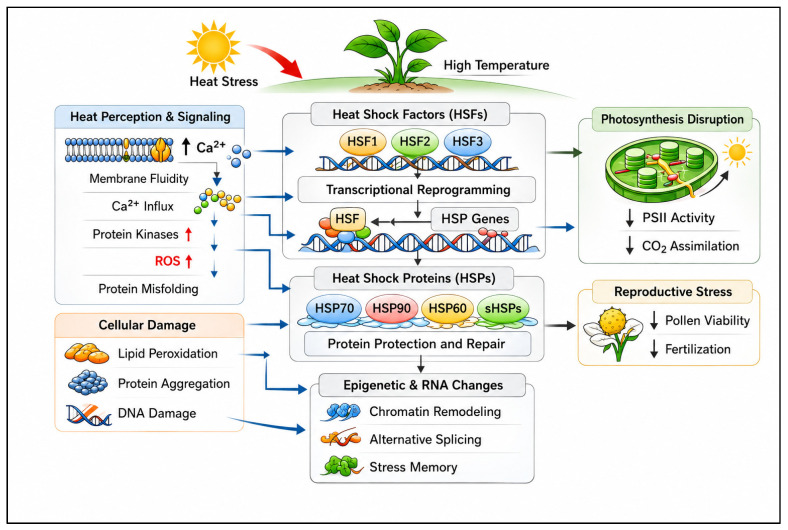
Schematic representation of plant responses to heat stress. Exposure to elevated temperatures destabilizes cellular membranes and proteins, leading to the accumulation of reactive oxygen species and the activation of stress signaling pathways. Heat perception triggers calcium-dependent signaling cascades and protein kinase activation, which in turn stimulate heat shock factors (HSFs). Activated HSFs drive transcriptional reprogramming and induce expression of heat shock proteins (HSPs), which act as molecular chaperones to prevent protein aggregation and maintain protein homeostasis [69,70]. Heat stress also impairs photosynthesis by disrupting chloroplast structure and inhibiting key enzymes involved in carbon fixation. Reproductive development is particularly vulnerable, with elevated temperatures reducing pollen viability and fertilization success. Prolonged or repeated exposure induces chromatin remodeling and changes in RNA processing, contributing to heat stress acclimation and the establishment of stress memory [68,76].

Heat stress has emerged as a major abiotic factor threatening plant survival and productivity, particularly amid accelerating global warming and the increasing frequency of extreme temperature events [62,63]. Exposure to elevated temperatures disrupts cellular integrity by destabilizing membranes, inducing protein misfolding, and increasing the accumulation of reactive oxygen species, collectively impairing metabolic coordination and cellular viability [64,65]. Heat perception relies on temperature-induced alterations in membrane dynamics and macromolecular structure, rapidly activating calcium-dependent signaling pathways and protein kinase cascades that initiate stress-responsive gene regulation [64,66]. A key molecular hallmark of heat stress is the activation of heat shock factors, which orchestrate large-scale transcriptional reprogramming by binding heat shock elements and inducing the expression of genes involved in protein quality control and cellular protection (Figure 4) [67,68]. This response is marked by a strong induction of heat-shock proteins (HSPs), which function as molecular chaperones, stabilizing unfolded proteins and preventing irreversible aggregation under thermal stress [69,70]. Different HSP families exhibit distinct but complementary functions. HSP70s are among the most extensively studied and act as ATP-dependent chaperones that assist in de novo protein folding and the refolding of stress-denatured polypeptides, thereby limiting aggregation and preserving protein functionality under heat stress conditions [71]. HSP90 proteins primarily function to stabilize and mature key regulatory proteins, including stress-responsive kinases and transcription factors, thereby contributing to the fine-tuning of stress signaling pathways [72]. In contrast, small heat shock proteins (sHSPs) operate in an ATP-independent manner, forming oligomeric complexes that bind partially unfolded proteins and maintain them in a refolding-competent state during acute thermal stress episodes [73].

Elevated temperatures also disrupt the photosynthetic machinery by impairing electron transport and inhibiting key enzymes of carbon fixation, linking molecular dysfunction to reduced growth and biomass accumulation [74]. Heat stress severely compromises reproductive success by altering gene expression programs required for pollen development and fertilization, leading to reduced seed set and yield instability [75]. Recurrent or prolonged heat exposure further induces persistent regulatory changes, including chromatin remodeling, alternative splicing, and stress-associated transcriptional memory, which enhance plants’ capacity to respond to subsequent heat episodes (Figure 4) [68,76].

## 3. Phytohormones

### 3.1. Abscisic Acid

Abscisic acid (ABA) is a carotenoid-derived sesquiterpenoid phytohormone whose biosynthesis is initiated in plastids through the oxidative cleavage of 9-cis-epoxycarotenoids by 9-cis-epoxycarotenoid dioxygenase (NCED) enzymes, thereby linking stress perception to ABA biosynthesis and accumulation [77]. The resulting molecule contains a cyclohexenone ring and a short acidic side chain, structural features essential for receptor binding and the dynamic regulation of its bioactive pool within plant tissues [19]. ABA accumulation under drought, salinity, cold, or osmotic stress activates a conserved signaling module centered on the pyrabactin resistance/pyrabactin resistance-like/regulatory component of the ABA receptor (PYR/PYL/RCAR) family of soluble receptors, which function as ligand-dependent inhibitors of clade A protein phosphatase 2Cs (PP2Cs) [78,79]. Upon ABA binding, these receptors undergo conformational changes that promote stable complex formation with PP2Cs, thereby relieving PP2C-mediated repression of SNF1-related protein kinases 2 (SnRK2s), which act as central signal transducers [80,81]. Activated SnRK2s phosphorylate multiple downstream targets, including ion channels and transcriptional regulators, enabling rapid and long-term stress responses to be coordinated within the same signaling framework [82]. One of the best-characterized outputs of this molecular cascade is the regulation of stomatal movement, where SnRK2-mediated phosphorylation modulates anion and potassium channels in guard cells, leading to membrane depolarization and stomatal closure [83]. This process is tightly integrated with secondary signaling molecules, such as Ca^2+^ and reactive oxygen species, which act both upstream and downstream of ABA perception to reinforce signal specificity and robustness [84].

Beyond immediate physiological adjustments, ABA signaling exerts profound control over gene expression by activating ABA-responsive transcription factors, including members of the bZIP, MYB, and NAC families, which bind conserved cis-elements in stress-inducible promoters [85,86]. This transcriptional reprogramming promotes the accumulation of protective proteins, including late embryogenesis abundant (LEA) proteins, enzymes involved in osmolyte biosynthesis, and components of antioxidant systems, collectively enhancing cellular stability under prolonged stress [87,88]. ABA signaling does not operate in isolation but is embedded within an extensive hormonal network, where it interacts with gibberellins, auxins, ethylene, and jasmonates at multiple regulatory nodes, including shared transcriptional targets and post-translational modifications of signaling proteins [89]. This crosstalk allows plants to balance growth suppression with stress tolerance, ensuring that energy resources are redirected toward survival without irreversibly arresting development [24].

### 3.2. Salicylic Acid

Salicylic acid (SA) is a low-molecular-weight phenolic phytohormone with a benzene ring bearing an ortho-hydroxyl group and a carboxylate group, features that underpin its biochemical activity in plants and enable interactions with diverse signaling proteins and redox partners [90]. SA is synthesized primarily via the isochorismate pathway in chloroplasts, and evidence suggests that alternative biosynthetic routes vary by species, indicating flexibility in its production that can affect downstream stress signaling [91]. SA functions as a central signaling molecule that modulates stress responses by altering transcriptional networks, regulating redox homeostasis, and influencing key stress-responsive genes [92].

Under abiotic stress, endogenous SA levels often rise, triggering the expression of antioxidant enzymes, heat shock proteins, and late embryogenesis abundant proteins that collectively enhance tolerance to drought, salinity, and temperature extremes [93]. SA’s action under stress involves ROS signaling, in which moderate increases in ROS, such as hydrogen peroxide (H_2_O_2_), serve as second messengers that amplify SA signaling cascades; this interplay modulates transcription factor activity and stress-gene promoters [94]. The hormone also interacts with MAPK cascades, Ca^2+^ signaling, and nitric oxide pathways, forming an integrated molecular network that fine-tunes gene expression and physiological responses under unfavorable conditions [95]. Importantly, SA engages in hormonal crosstalk with abscisic acid, jasmonates, and auxin, enabling plants to coordinate growth and stress adaptation by modulating both stress-tolerance mechanisms and developmental programs at the transcriptional level [96].

Exogenous SA applications have been shown to confer enhanced resilience to abiotic stress by activating specific transporters, stabilizing cellular membranes, and upregulating protective metabolites, thereby emphasizing its role as a signaling hub rather than merely a metabolic intermediate [97].

### 3.3. Jasmonate

Jasmonates are oxylipin-derived phytohormones that play a central role in regulating plant growth, development, and stress adaptation, particularly under adverse environmental conditions [98]. These compounds are synthesized from α-linolenic acid via the octadecanoid pathway, a series of enzymatic reactions initiated in chloroplast membranes and completed in peroxisomes [99]. Structurally, jasmonates feature a cyclopentanone ring essential for biological activity and recognition, enabling them to function as potent signaling molecules in plants [100]. Among the forms, jasmonic acid and its methylated and amino acid-conjugated derivatives are the most biologically relevant jasmonates involved in stress signaling [101]. After biosynthesis, jasmonic acid is converted to jasmonoyl-isoleucine (JA-Ile), the principal bioactive ligand in jasmonate signaling pathways [102]. JA-Ile is perceived by the Skp1–Cullin–F-box Coronatine-Insensitive1 (SCFCOI1) receptor complex, triggering degradation of Jasmonate ZIM-domain (JAZ) repressor proteins via the ubiquitin–proteasome system and enabling activation of jasmonate-responsive transcription factors, including the basic helix–loop–helix (bHLH) transcription factor MYC2 [103]. In the absence of stress, JAZ proteins suppress jasmonate-regulated genes by physically interacting with transcription factors, thereby maintaining growth-related processes [104].

Environmental stimuli rapidly increase endogenous jasmonate levels, triggering transcriptional reprogramming that prioritizes defense and stress tolerance overgrowth [105]. Jasmonates are strongly linked to plant responses to abiotic stress, including drought, salinity, temperature extremes, and heavy metal toxicity [106]. Under drought conditions, elevated jasmonate accumulation promotes stomatal regulation, antioxidant enzyme activity, and osmolyte production, thereby reducing cellular damage caused by water deficit [107].

Salt stress also induces jasmonate signaling, which contributes to ion homeostasis, membrane stability, and enhanced stress tolerance by regulating stress-responsive genes [108]. Similarly, exposure to low temperatures activates jasmonate-dependent pathways that support membrane fluidity and enhance tolerance to chilling and freezing stress [109]. Jasmonate signaling does not operate in isolation but interacts extensively with other phytohormones, including abscisic acid, salicylic acid, and ethylene, forming an integrated regulatory network that balances growth and stress responses [110].

### 3.4. Gibberellin

Gibberellins (GAs) regulate key developmental processes, including stem elongation, seed germination, flowering, and fruit development. These effects are mediated by changes in gene expression and protein activity in response to hormonal cues [111]. The primary GA receptor in higher plants is Gibberellin Insensitive Dwarf1 (GID1). This soluble protein binds bioactive gibberellin (GA) and undergoes a conformational change that promotes interaction with growth repressors known as DELLA proteins, which act as negative regulators of GA responses [112]. Upon GA binding to GID1, the GA–GID1 complex recruits DELLA proteins and facilitates their recognition by the SKP1–CULLIN–F-BOX E3 ubiquitin ligase complex (SCF–E3), leading to DELLA ubiquitination and degradation by the 26S proteasome, thereby relieving repression of downstream growth-related regulators [113].

DELLA proteins regulate the availability of transcription factors that promote cell expansion and division by inhibiting their activity until GA-induced DELLA degradation occurs, thereby promoting elongation and developmental transitions [114]. Under drought, salinity, and cold stress, plants exhibit reduced endogenous gibberellin levels due to increased expression of Gibberellin 2-Oxidase (GA2ox) genes and decreased expression of gibberellin biosynthetic genes, leading to the accumulation of DELLA repressors and moderated growth [115]. Suppressed GA signaling under stress is associated with enhanced stress tolerance, as reduced growth conserves energy and resources that can be redirected toward protective processes such as osmotic adjustment and antioxidant defense [116]. Specific transcription factors from the Apetala2/Ethylene Responsive Factor (AP2/ERF) and dehydration-responsive element-binding/C-repeat binding factor (DREB/CBF) families regulate GA metabolic genes under cold and osmotic stress, linking environmental sensing to hormonal regulation and influencing DELLA accumulation [117].

Interactions between gibberellin signaling and other hormonal pathways, including ABA, JA, and ET, influence stress responses through shared regulators and reciprocal effects on gene expression, thereby contributing to adaptive stress outcomes [118]. Exogenous application of gibberellins to stressed plants can mitigate stress effects by enhancing antioxidant enzyme activity, modulating hormonal balance, and improving photosynthetic efficiency, underscoring GA’s role in stress modulation [119].

### 3.5. Auxin

Auxin is a major class of plant phytohormones characterized by an indolic backbone, with indole-3-acetic acid (IAA) as the predominant naturally occurring form, synthesized primarily in young tissues [120]. Its small, aromatic structure enables directional transport via specialized transport systems, including AUX1/LAX influx carriers and PIN-FORMED (PIN)-type efflux proteins, thereby generating spatial hormone gradients that guide developmental processes [121]. Auxin perception relies on a nuclear co-receptor system in which Transport Inhibitor Response1/Auxin Signaling F-Box (TIR1/AFB) proteins bind auxin and promote the ubiquitination and degradation of Aux/IAA repressors [122,123].

The removal of these repressors allows Auxin Response Factor (ARF) proteins to bind auxin-responsive cis-elements in target promoters, thereby modulating transcriptional outputs in a context-dependent manner [124]. Beyond this canonical pathway, auxin can also trigger rapid cellular responses through alternative signaling routes involving membrane-associated kinases, enabling plants to respond swiftly to environmental cues without extensive transcriptional reprogramming [125].

Auxin availability is further regulated by conjugation, oxidative inactivation, and feedback control of biosynthetic enzymes, ensuring precise adjustment of hormone levels under fluctuating conditions [126]. Exposure to abiotic stresses such as drought and salinity disrupts auxin distribution, leading to pronounced changes in root architecture and growth dynamics [127]. Stress-induced changes in the abundance and polarity of PIN transporters reshape auxin maxima within root tissues, contributing to adaptive responses, including suppressed lateral root formation and modified elongation zones [128].

Specific members of the ARF and Aux/IAA families exhibit stress-responsive expression profiles, linking auxin signaling networks to broader regulatory programs activated under adverse environmental conditions [129]. In parallel, small regulatory RNAs modulate the stability and translation of auxin-related transcripts, adding flexibility and precision to stress-associated hormonal regulation [130,131]. Auxin does not act in isolation during stress adaptation but interacts extensively with other hormonal pathways, particularly ABA and ET, enabling coordinated control of growth, restraint, and survival strategies [132].

### 3.6. Ethylene

Ethylene (C_2_H_4_) is a small, unsaturated hydrocarbon with two carbon atoms linked by a double bond in a planar configuration. This structural simplicity underlies its rapid diffusion and gaseous signaling properties in plants [133]. Because it is a gas at physiological temperatures, ethylene readily diffuses across membranes and through intercellular spaces without specialized transport systems, enabling coordinated developmental and stress responses [134]. Ethylene biosynthesis follows the Yang cycle, in which S-adenosyl-L-methionine (SAM) is converted to 1-aminocyclopropane-1-carboxylic acid (ACC), which is then oxidized to ethylene [135]. ACC synthase (ACS), the rate-limiting enzyme of this pathway, is encoded by a multigene family, and its transcription is strongly induced by environmental constraints such as salinity, drought, and temperature extremes [136]. Beyond transcriptional control, ACS protein stability is modulated by phosphorylation and ubiquitin-mediated degradation, providing an additional regulatory layer during stress adaptation [137,138].

Ethylene perception occurs primarily at the endoplasmic reticulum membrane via receptors such as ethylene response factor 1 (ETR1), which act as negative regulators in the absence of the hormone [139]. Upon hormone binding, receptor inactivation suppresses the Raf-like kinase constitutive triple response 1 (CTR1), thereby permitting signal propagation to downstream components [140]. This signaling cascade culminates in the activation of ethylene-insensitive 2 (EIN2), whose C-terminal domain relays the signal to the nucleus, stabilizing ethylene-insensitive 3/ethylene-insensitive-like (EIN3/EIL) transcription factors [141,142]. EIN3 and related transcription factors subsequently regulate large sets of ethylene-responsive genes, including members of the ethylene response factor (ERF) family that coordinate stress-related transcriptional programs [143]. Under salinity stress, ethylene signaling contributes to ionic homeostasis and enhances antioxidant capacity, partly through transcriptional activation of ROS-scavenging enzymes [144,145].

During drought, ethylene interacts dynamically with ABA, influencing stomatal closure and modulating root growth to optimize water acquisition [146]. Ethylene also regulates auxin biosynthesis and polar transport under stress, thereby reshaping root system architecture in response to adverse soil conditions [147,148]. Heat stress stimulates ethylene production, which in turn modulates heat shock protein expression and cellular protein-folding homeostasis [149]. Transcriptomic and proteomic analyses indicate that ethylene signaling integrates with calcium-dependent pathways, mitogen-activated protein kinase (MAP kinase) cascades, and redox networks during abiotic stress acclimation [150].

### 3.7. Melatonin

Melatonin (N-acetyl-5-methoxytryptamine) is an indoleamine derived from tryptophan. Its amphiphilic structure enables diffusion across biological membranes and facilitates its distribution among subcellular compartments in plant cells [151]. In higher plants, its biosynthesis proceeds through tryptamine and serotonin intermediates, involving tryptophan decarboxylase, tryptamine 5-hydroxylase, serotonin N-acetyltransferase, and acetylserotonin O-methyltransferase, thereby linking melatonin production to primary amino acid metabolism [152,153]. The structural resemblance between melatonin and indole-3-acetic acid has prompted discussion of shared precursors and partial overlap in developmental regulation, particularly in root architecture and shoot growth responses [154].

Experimental evidence shows that melatonin accumulation rises rapidly under drought and salinity stress, during which it acts as a direct scavenger of hydroxyl radicals, hydrogen peroxide, and superoxide anions, thereby limiting oxidative injury to membranes and macromolecules [155]. Genetic and biochemical analyses further show that exogenous or endogenous melatonin enhances the activity and transcription of antioxidant enzymes, including superoxide dismutase, catalase, and ascorbate peroxidase, during stress [156,157]. Cold- and heat-stress studies reveal that melatonin preserves chlorophyll content, stabilizes photosystem II efficiency, and sustains carbon assimilation rates, thereby maintaining energy balance under temperature extremes [158,159,160]. Research in Arabidopsis has identified a candidate G protein-coupled receptor 2/phytomelatonin receptor 1 (CAND2/PMTR1) as a melatonin receptor involved in stomatal regulation, suggesting that melatonin perception can influence guard cell signaling and downstream stress responses [161].

Transcriptomic profiling under salinity and heavy-metal exposure indicates that melatonin treatment reshapes the expression of genes involved in redox homeostasis, ion transport, osmolyte biosynthesis, and secondary metabolism, thereby supporting coordinated metabolic reprogramming in response to environmental challenges [162].

### 3.8. Strigolactone

Strigolactones (SLs) are carotenoid-derived terpenoid lactones that function as endogenous regulators of plant architecture and environmental adaptation [163,164]. Structurally, canonical SLs consist of a tricyclic ABC scaffold linked via an enol-ether bridge to a methylbutenolide (D-ring). In contrast, non-canonical SLs retain the conserved bioactive D-ring but lack the complete ABC moiety [165,166]. SL biosynthesis begins with all-trans-β-carotene isomerization catalyzed by DWARF27 (D27), followed by sequential oxidative cleavage by carotenoid cleavage dioxygenases 7 (CCD7) and 8 (CCD8), producing carlactone as a central intermediate. Carlactone is subsequently converted into diverse SL derivatives through oxidation reactions catalyzed by cytochrome P450 monooxygenases of the MORE AXILLARY GROWTH1 (MAX1) family [167,168]. SL perception and signaling are mediated by the α/β-hydrolase receptor DWARF14 (D14). Upon binding and hydrolysis of SLs within its catalytic pocket, D14 undergoes a conformational change that promotes interaction with the F-box protein MORE AXILLARY GROWTH2 (MAX2, also known as D3) [169,170]. This ligand-dependent modification stabilizes D14 in an active state, thereby enabling assembly of the Skp1–Cullin–F-box (SCF^MAX2) ubiquitin ligase complex [171]. The SCF^MAX2 complex targets SUPPRESSOR OF MAX2 1-LIKE (SMXL) proteins, including DWARF53 (D53), for ubiquitination and degradation via the 26S proteasome, thereby relieving transcriptional repression and activating downstream gene expression programs [172]. SMXL proteins are known to recruit TOPLESS co-repressors and chromatin remodeling factors, indicating that SL signaling regulates gene expression through direct modulation of transcriptional repression and chromatin accessibility [173,174]. Under drought and salinity stress, SL biosynthetic genes (D27, CCD7, CCD8, and MAX1) are transcriptionally upregulated, leading to increased endogenous SL accumulation [175]. Elevated SL levels enhance the degradation of SMXL repressors, which normally inhibit stress-responsive transcriptional programs, thereby facilitating stress adaptation at the transcriptional level [172]. In wheat, treatment with the synthetic SL analog GR24 reduces oxidative damage markers such as hydrogen peroxide and malondialdehyde, while increasing antioxidant enzyme activities and osmotic adjustment capacity, indicating that SLs contribute to stress tolerance at both biochemical and molecular levels [176]. Hormonal crosstalk during stress adaptation further integrates SL signaling with other pathways. In the case of abscisic acid (ABA), SL signaling modulates ABA sensitivity through MAX2-dependent regulation of ABA signaling components, including effects on SnRK2 kinase activity and ABA-responsive transcription factors [177]. Additionally, SL signaling contributes to reactive oxygen species (ROS) homeostasis by regulating antioxidant genes encoding superoxide dismutase, catalase, and peroxidases downstream of SMXL degradation [178]. Under osmotic stress, SL signaling also influences root system architecture by modulating auxin transport through regulation of PIN-FORMED (PIN) auxin efflux carriers, thereby altering auxin distribution patterns to optimize water uptake [179]. Recent structural and biochemical evidence indicates that MAX2 also participates in karrikin signaling via the related receptor KARRIKIN INSENSITIVE2 (KAI2), highlighting that SL signaling components integrate multiple environmental cues through shared ubiquitination machinery [180,181].

## 4. Phytohormone Crosstalk in Abiotic Stress

Plant responses to abiotic stresses, including drought, salinity, extreme temperatures, and nutrient deprivation, are governed by a highly interconnected hormonal network rather than by linear signaling pathways acting independently [24]. Growing evidence indicates that abscisic acid, salicylic acid, gibberellins, auxin, ethylene, jasmonates, strigolactones, and melatonin function within a dynamic regulatory framework in which signals integrate at multiple molecular levels, including biosynthesis, receptor activation, protein phosphorylation cascades, transcriptional control, and targeted protein degradation [23,182].

Plant hormonal crosstalk during abiotic stress can be broadly categorized into synergistic, antagonistic, and hierarchical interactions, each contributing distinctly to stress adaptation and developmental plasticity [183]. Synergistic interactions occur when two or more phytohormones cooperatively enhance stress-responsive pathways; for example, abscisic acid (ABA) and jasmonates (JAs) frequently act in concert to promote stomatal closure, antioxidant defense, and osmotic adjustment under drought and salinity conditions [106]. In contrast, antagonistic interactions involve the suppression of one hormonal pathway by another, thereby balancing growth and defense priorities. A well-established example is the antagonism between ABA and gibberellins (GAs), which coordinates seed germination, dormancy, and stress-induced growth inhibition [184]. Similarly, salicylic acid (SA) and auxin often exert opposing effects on stress adaptation and developmental regulation [185]. Beyond synergistic and antagonistic relationships, phytohormonal signaling is also organized hierarchically, in which certain hormones act upstream to regulate the biosynthesis, transport, perception, or signaling competence of other hormonal pathways. ABA, for instance, frequently acts as a central upstream regulator during abiotic stress, modulating ethylene and auxin responses through interconnected transcriptional and post-translational mechanisms [23]. Plant adaptation to abiotic stress depends not only on the individual functions of hormones but also on a dynamic balance between growth-promoting and stress-responsive signaling pathways. This hormonal balance determines the allocation of resources between growth maintenance and stress defense, ultimately defining the physiological outcome under adverse environmental conditions [182].

**Figure 5:**
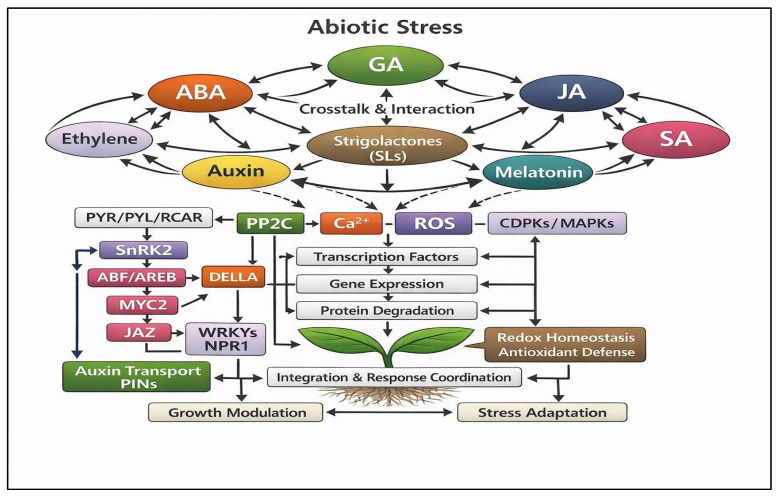
Integrated hormonal network regulating plant responses to abiotic stress. Stress signals activate ABA signaling through the PYR/PYL/RCAR–PP2C–SnRK2 module, triggering ABF/AREB-dependent transcriptional reprogramming. Extensive crosstalk occurs among ABA and other hormones, including GA (via DELLA stabilization), JA (through DELLA–JAZ–MYC2 interactions), SA, ethylene, auxin, strigolactones, and melatonin [186,187]. Signal integration is mediated by shared components such as ROS, Ca^2+^, CDPK/MAPK cascades, and ubiquitin-dependent protein degradation [190]. These multilayered interactions coordinate growth modulation, redox homeostasis, and stress adaptation under abiotic stress.

This multilayered crosstalk enables plants to coordinate growth modulation with stress acclimation, thereby optimizing survival under fluctuating environmental conditions [89]. Under water deficit or high salinity, ABA rapidly accumulates and serves as a primary signal that initiates adaptive responses. At the molecular level, ABA perception by PYR/PYL/RCAR receptors inhibits clade A PP2C phosphatases, thereby activating SnRK2 kinases and phosphorylating downstream transcription factors, including ABF/AREB proteins (Figure 5) [78,79].

However, the output of this core module is extensively reshaped by interactions with other hormonal pathways. For example, ABA-mediated stabilization of DELLA proteins under stress conditions links ABA signaling to GA repression, as elevated ABA levels suppress GA biosynthesis. At the same time, DELLA accumulation restrains expression of growth-related genes [186].

DELLAs, in turn, physically interact with JAZ repressors of the JA pathway, thereby modulating MYC2-dependent transcription and creating a regulatory interface among ABA, GA, and JA during stress adaptation (Figure 5) [110,187]. Through these interactions, growth inhibition and defense activation become tightly coordinated rather than mutually exclusive [188]. Signal convergence is also evident in shared transcription factors and second messengers. MYC2 is a central node that integrates ABA and JA signals, as it is activated downstream of SnRK2 kinases and released from JAZ-mediated repression during JA signaling [189].

Similarly, ROS generated by NADPH oxidases serve as common intermediates in ABA- and ET-mediated stomatal regulation. CDPKs and MAPK cascades also provide additional points for signal amplification and integration (Figure 5) [190]. Ethylene can modulate ABA sensitivity in guard cells through ROS-dependent mechanisms, indicating that hormonal interactions often occur downstream of receptor activation, mediated by shared signaling components rather than at the level of biosynthesis alone [191]. Auxin signaling, which primarily regulates cell elongation and developmental patterning, is also extensively reprogrammed during abiotic stress [127].

ABA influences auxin distribution by altering the expression and subcellular localization of PIN efflux carriers, thereby reshaping root system architecture under drought or salinity [192]. Moreover, stress-induced stabilization of DELLA proteins interferes with auxin-responsive transcription by interacting with the AUX/IAA and ARF modules, illustrating how growth-promoting signals are attenuated in favor of stress resilience [193]. This antagonistic yet coordinated relationship between auxin and ABA ensures that developmental plasticity is maintained while preventing excessive growth under unfavorable conditions [194]. Crosstalk between SA and other hormones further complicates stress signaling networks.

Although SA is traditionally associated with biotic stress responses, it also contributes to abiotic stress tolerance by modulating antioxidant systems and redox homeostasis [90]. Interactions between SA and ABA are often antagonistic, mediated in part by NPR1-dependent transcriptional regulation and changes in cellular redox status [195]. At the same time, JA–SA antagonism, mediated by transcription factors such as WRKYs and MYC2, influences oxidative stress responses under abiotic constraints (Figure 5) [98]. These regulatory modules illustrate how stress-specific outputs are shaped by competitive or cooperative transcriptional control. Strigolactones and melatonin have emerged as additional components of this regulatory web. Strigolactones, perceived via the D14 receptor and MAX2-dependent ubiquitination pathways, interact genetically and functionally with ABA signaling to enhance drought tolerance and modulate stomatal behavior [175]. They also influence auxin transport dynamics, thereby indirectly contributing to the ABA–auxin balance during stress-induced architectural adjustments [196].

Beyond its well-documented antioxidant properties, melatonin modulates the expression of genes involved in ABA biosynthesis, JA signaling, and auxin transport, suggesting that it serves as a fine-tuning regulator within broader hormonal networks [197]. Through transcriptional reprogramming and ROS scavenging, melatonin helps maintain hormonal equilibrium under prolonged stress (Figure 5) [198].

## 5. Conclusions

We have made significant progress in elucidating the complex mechanisms by which plants perceive and respond to diverse abiotic stresses. Phytohormones, including abscisic acid, salicylic acid, jasmonates, gibberellins, auxin, ethylene, melatonin, and strigolactones, function not as isolated regulators but as components of highly interconnected signaling networks. These networks coordinate physiological, biochemical, and molecular responses, enabling plants to balance growth, development, and survival under adverse environmental conditions such as drought, salinity, and extreme temperatures. Hormonal crosstalk has emerged as a central feature of these networks, allowing plants to integrate multiple stress signals, fine-tune adaptive responses, and maintain cellular homeostasis. The growing understanding of key molecular players, such as receptor complexes, transcription factors, reactive oxygen species, and protein kinase cascades, provides a foundation for targeted strategies to enhance stress tolerance.

## 6. Future Perspectives and Challenges

Despite major advances, the regulation of abiotic stress responses by phytohormones remains incompletely understood, particularly under combined stress conditions that better reflect natural environments. Most current knowledge is still derived from single-stress experiments, limiting our understanding of how hormonal networks behave under complex field-like scenarios. In addition, the lack of spatial and temporal resolution in hormone signaling studies remains a key limitation, as hormonal dynamics vary significantly across tissues and developmental stages. Emerging imaging technologies and genetically encoded biosensors may help address this challenge by enabling real-time monitoring of hormone distribution and signaling activity in planta. Although core components of phytohormone signaling networks have been identified, the mechanisms by which multiple hormonal pathways are integrated, prioritized, and dynamically reconfigured under stress conditions remain insufficiently resolved. Future research should therefore emphasize systems-level and multi-omics approaches to capture the complexity of hormonal crosstalk and its role in stress adaptation.

Importantly, increasing mechanistic knowledge of phytohormone signaling also provides a strong foundation for crop improvement strategies. Because phytohormones regulate interconnected processes involved in stress perception, growth regulation, antioxidant defense, osmotic adjustment, and developmental plasticity, their signaling networks represent attractive targets for engineering stress-resilient crops [23]. Recent advances in CRISPR/Cas-based genome editing, molecular breeding, pathway engineering, and high-throughput phenotyping now enable precise manipulation of key regulatory nodes, including hormone biosynthesis enzymes, receptors, transporters, transcription factors, and downstream signaling components [199,200]. Rather than broadly altering hormonal systems, these approaches aim to fine-tune specific signaling modules to optimize stress adaptation. For example, recent CRISPR/Cas-based genome editing strategies targeting negative regulators of ABA signaling, particularly PP2C family members, have generated promising drought-tolerant phenotypes in several crop species without causing major developmental penalties. Moreover, fine-tuning ABA sensitivity, rather than constitutive pathway activation, is increasingly viewed as a critical strategy for minimizing growth–stress trade-offs [201,202].

In the near future, integrating multi-omics datasets with precision genome editing and advanced phenotyping platforms is expected to further accelerate functional discovery and trait engineering. These integrative approaches will enhance our ability to decode dynamic hormonal interactions under combined stress and facilitate the rational design of climate-resilient crop varieties that maintain productivity amid increasingly variable environmental conditions.

## Data Availability

No new data were created or analyzed in this study.
